# Miscellaneous

**Published:** 1887-04

**Authors:** 


### MISCELLANEOUS.

A STATUE recently discovered in the bed of the Tiber proves to be a Bacchus. He stands six feet high, is cast in bronze, with ivory eyes, is exquisitely modelled, and in excellent preservation.

THE megaroscope is a new apparatus lately brought to the notice of the Académic des Sciences. It is armed with an incandescent light, and is used to explore the stomach, bladder, and other cavities of the body. By its means an objective lens may be carried into the cavity and a magnified image of its interior be obtained for the physician's inspection.

THE great glacier of Alaska is moving at the rate of a quarter of a mile per annum toward the sea. The front presents a wall of ice some 500 feet thick, its breadth varies from 3 to 10 miles, and it is about 150 miles long. Almost every quarter of an hour hundreds of tons of ice in large blocks fall into the sea, which they agitate in the most violent manner. The ice is extremely pure and has tints of the lightest blue as well as the deepest indigo. The top is very broken, forming small hills, and even chains of mountains in miniature.

IN one of the German health-resorts, the experiment was tried, last summer, of having the patients with pulmonary disorders sleep all night in the open air in the pine woods. The hammocks, used to rest in during the day, were provided with pillows and bed-clothing, and a party of five, two ladies and three gentlemen, spent their nights in the woods with no roof over their heads. The experiment was very successful, the patients slept better than they had been able to do in their rooms, and all declared themselves as feeling much more refreshed by their sleep than usual. It is proposed next summer to provide accommodation for a large number of patients in the forest, so that the experiment may be tried on a large scale.

HE OWNED ENOUGH.—At one of the stations on the San Francisco and Northern Pacific Railway, a few Sundays since, an elderly gentleman got off the cars to take brief observations during the stoppage of the train. The assistant at the station rushed out and made a regular baggage smasher's attack on a trunk, which he slammed about with a reckless disregard of consequences. The other man interposed ; "Young man, won't you break that trunk ?" The young man turned a withering look upon the old gentleman and impudently inquired : "What's the matter with you ? Do you own this trunk ?" "No, sir ?" came back in a tone that evinced much indignation, "but I'll have you to understand, sir, that I own this railroad." As Col. Donahue moved back to the train the limp young man reclined against the station for support.—*Healdsburg Enterprise.*

"YES, Satan is let loose upon the earth, he wears the garb of the nineteenth century, he is found in all public places, he is the type of modern society, he is found wherever human pride and passion prevail, he upholds kingdoms, he sustains monarchies, he believes in established churches, he knows that authority is best, he tempts mankind by worldly possessions, and wins them unto his estate by all kinds of allurements ; he *is found in the sanctioned selfishness of the world* all over the habitable globe, Do not think because you have vanquished the Satan of theology that you have overcome the Satan of the world. Look within. There you will find that, though the Satan of theology were slain thousands and thousands of times, you have still to conquer this Satan of worldliness who walks in your midst. Until you see to it that the strong do not trample upon the weak, that the ignorant are not ground down to ignorance, that the powerless are not tethered to the narrow limits of their daily toil, then you are full in the wake of Satan."—*Cora L. V. Richmond.*

Clairvoyance.—Our beautiful boy, nearly five years old, was taken sick ; able to be about for ten days or two weeks, when he had a spasm, said to have been a worm fit. From time to time, for about six weeks, he was under the care of three fairly intelligent and successful physicians neither of whom gave any definite diagnosis of the case, or emphatic agreement as to import of symptoms. A lady, then member of our family, asked for a lock of his hair. She sent it to a lady in Chicago, not a regular physician, saying, "The little boy where I am staying is sick ; can you tell us what is the matter ?" By return mail came the reply : "He has effusion of water on the brain, and will not get well." A little time afterward, one eye turned. By what capacity of mind or soul did the lady, not a regular physician, diagnose the case correctly one hundred and sixty miles distant, that three fairly intelligent physicians failed to discover, though with him from time to time for at least two months ?—*J. R. Tallmadge in Mind in Nature.*

Coking the Dead.—A new sort of cremation, or rather incineration, has been produced by a gas engineer of Monongahela, by which it is said the solid and volatile portions of bodies are separated, the former into a mummy of coke, and the latter into gas. The remains are not reduced to ashes, but will be returned to friends, to stand up in the corner or keep in any shape that their tastes may prefer. A dog was killed and brought to the gas-works, and the retort being heated to a proper degree, the body was shoved in and the caps screwed on. The gas gauge was watched and it was found that while the dog was incinerated he made 180 feet of gas. In the course of a couple of hours the retort was opened. The frame of the dog was whole, being formed into a perfect piece of coke, which could be handled with no more danger of breaking than an ordinary piece of coked coal, and when removed but a very slight odor was emitted from the retort. —*Special to Chicago Tribune.*

### A RIDDLE.

'Tis said that Felix always keeps his word,
Though oft and oft I've known the fellow break it ;
The riddle's plain, if rightly understood,
*He keeps his word, because no one will take it.*

